# 4C3 Human Monoclonal Antibody: A Proof of Concept for Non-pathogenic Proteinase 3 Anti-neutrophil Cytoplasmic Antibodies in Granulomatosis With Polyangiitis

**DOI:** 10.3389/fimmu.2020.573040

**Published:** 2020-09-25

**Authors:** Jérôme Granel, Roxane Lemoine, Eric Morello, Yann Gallais, Julie Mariot, Marion Drapeau, Astrid Musnier, Anne Poupon, Martine Pugnière, Seda Seren, Dalila Nouar, Valérie Gouilleux-Gruart, Hervé Watier, Brice Korkmaz, Cyrille Hoarau

**Affiliations:** ^1^Plateforme B Cell Ressources (BCR) EA4245, Université de Tours, Tours, France; ^2^Service transversal d’Immunologie Clinique et d’Allergologie, Centre Hospitalier Régional Universitaire, Tours, France; ^3^MAbSilico SAS, Domaine de l’Orfasière, Nouzilly, France; ^4^Physiologie de la Reproduction et des Comportements, INRA UMR 0085, CNRS UMR 7247, Université de Tours, Tours, France; ^5^Institut de Recherche en Cancérologie, Institut Régional du Cancer, INSERM U1194, Université Montpellier, Montpellier, France; ^6^Centre d’Etude des Pathologies Respiratoires, INSERM, UMR 1100, Tours, France; ^8^Université de Tours, Tours, France; ^8^Laboratoire d’Immunologie, Centre Hospitalier Régional Universitaire, Tours, France

**Keywords:** anti-neutrophil cytoplasmic antibodies, proteinase 3, granulomatosis with polyangiitis, epitope, human neutrophils

## Abstract

Granulomatosis with polyangiitis (GPA) is a severe autoimmune vasculitis associated with the presence of anti-neutrophil cytoplasmic antibodies (ANCA) mainly targeting proteinase 3 (PR3), a neutrophilic serine proteinase. PR3-ANCA binding to membrane-bound PR3 on neutrophils induce their auto-immune activation responsible for vascular lesions. However, the correlation between PR3-ANCA level and disease activity remains inconsistent, suggesting the existence of non-pathogenic PR3-ANCA. In order to prove their existence, we immortalized B lymphocytes from blood samples of GPA patients in remission having persistent PR3-ANCA to isolate non-activating PR3-ANCA. We obtained for the first time a non-activating human IgG1κ anti-PR3 monoclonal antibody (mAb) named 4C3. This new mAb binds soluble PR3 with a high affinity and membrane-bound PR3 on an epitope close to the PR3 hydrophobic patch and in the vicinity of the active site. 4C3 is able to bind FcγRIIA and FcγRIIIB and has a G2F glycosylation profile on asparagine 297. 4C3 did not induce activation of neutrophils and could inhibit human polyclonal PR3-ANCA-induced activation suggesting that 4C3 is non-pathogenic. This characteristic relies on the recognized epitope on PR3 rather than to the Fc portion properties. The existence of non-pathogenic PR3-ANCA, which do not activate neutrophils, could explain the persistence of high PR3-ANCA levels in some GPA patients in remission and why PR3-ANCA would not predict relapse. Finally, these results offer promising perspectives particularly regarding the understanding of PR3-ANCA pathogenicity and the development of new diagnostic and therapeutic strategies in GPA.

## Introduction

Granulomatosis with polyangiitis (GPA) is a relatively rare necrotizing autoimmune systemic vasculitis affecting mainly small to medium vessels, with histological inflammatory lesions and granulomas (1, 2). Despite current therapies, this vasculitis can be severe and lethal (3). In vasculitis nomenclature, it is part of the group of anti-neutrophil cytoplasmic antibody (ANCA) associated vasculitis (AAV): GPA is associated with cytoplasmic ANCA (cANCA), detected by immunofluorescence (IF) on fixed neutrophils (1). These cANCA mainly target proteinase 3 (PR3) and are thus called PR3-ANCA. PR3 is a 29 kDa serine protease contained in neutrophils granules which can also be located at the membrane (mbPR3) (4). PR3-ANCA are very specific to GPA when detected by immunoassays (over 90%) (5). They are found in about three quarters of GPA patients, however, 10% of patients have MPO-ANCA and less than 10% have no detectable ANCA (5, 6).

Proteinase 3-ANCA have a central role in GPA pathophysiology: they bind neutrophils previously primed by tumor necrosis factor alpha (TNFα; called primed-neutrophils) and cause their auto-immune activation, which is responsible for vasculitis lesions (7, 8). Interaction between PR3-ANCA and neutrophils is of two types: on the one hand, a link between PR3-ANCA fragment antigen binding (Fab) and mbPR3 on primed neutrophils; and on the other hand, a link between PR3-ANCA fragment crystallisable (Fc) and Fc gamma receptors (FcγR); FcγRIIA (CD32a); and FcγRIIIB (CD16b) (9). Neutrophil activation by PR3-ANCA results in an adherence phenotype with increased expression of CD11b/CD18 (Mac-1) (10), induction of NETosis (11), production of reactive oxygen species (ROS) (12), protease release by degranulation (13) and production of proinflammatory cytokines (14).

Despite the central role of PR3-ANCA in GPA physiology, the correlation between PR3-ANCA level and disease activity is inconsistent in the literature (15, 16) except to predict relapse in patients with renal involvement (17, 18). Furthermore, PR3-ANCA can persist in GPA patients during remission without predicting relapse (16, 19), can be found in healthy people (20), and in other conditions than AAV (21). In a recent study, 15% of patients in complete remission had persistently positive PR3-ANCA > 12 months (16). Indeed, several factors involved in PR3-ANCA interaction with neutrophils (level of mbPR3, epitopes on PR3, subclass, and glycosylation of PR3-ANCA) influence auto-immune activation of neutrophils *in vitro* and disease activity. The level of mbPR3 correlates with neutrophil activation induced *in vitro* by PR3-ANCA and disease activity in GPA patients (9). PR3-ANCA targets different epitopes on PR3 with proportions differing between patients and with a different evolution in the same patient depending on disease activity (21, 22). PR3-ANCA found in the active phase of the disease mostly recognized a region close to the active site, corresponding to epitope 1 determined by using murine anti-human PR3 antibodies (23–25), and inhibit PR3 enzymatic activity *in vitro* (26–28). Furthermore, alpha 1-antitrypsin (α1AT), a natural inhibitor of PR3, removes induced-mbPR3 from the neutrophil membrane and through a conformational modification, impairs the binding of PR3 to PR3-ANCA-recognizing epitope 1 (29). Concerning isotypes of PR3-ANCA, IgG appears to be the most involved in PR3-ANCA pathogenicity, essentially IgG1 and IgG3 subclasses (14, 29, 30). Finally, glycosylation of PR3-ANCA linked to asparagine 297 is also involved in PR3-ANCA pathogenicity (31–33).

Therefore, these clinical and experimental data suggest the existence of non-pathogenic PR3-ANCA especially in patients in remission who have persistent PR3-ANCA and in healthy people. In this context, we aimed to obtain and characterize non-pathogenic PR3-ANCA from GPA patients in remission. In this study, after B cell immortalization, we presented a human monoclonal antibody (mAb) specific to PR3 (4C3) with a non-pathogenic function *in vitro*. Our results offer promising perspectives particularly concerning the understanding of PR3-ANCA pathogenicity in GPA and the development of new diagnostic and therapeutic strategies targeting non-pathogenic and pathogenic PR3-ANCA.

## Materials and Methods

### Obtaining PR3-ANCA From Immortalized B Cells of GPA Patients

#### Immortalization of B Cells From GPA Patients

Blood samples were obtained from GPA patients treated at the Regional University Hospital Center of Tours after informed consent and from healthy donors of the French Blood Establishment Centre-Atlantique. The samples were registered in a collection of human biological samples reported to the Ministry of Research (DC-2012-1636) in accordance with Decree N°2007-1120 of August 2007. Briefly, blood samples from GPA patients in remission with a persistent PR3-ANCA level (Table 1) were collected on Acid-Citrate-Dextrose tubes. Peripheral Blood Mononuclear Cells (PBMC) were then isolated using density gradient centrifugation (Lymphosep, Biowest, France) and enriched with memory B lymphocytes after isolation of total B lymphocytes with the «B-cell isolation kit» (Miltenyi Biotech, Germany) followed by cell sorting of memory B cells (CD19^+^ CD27^+^) with FACS Melody (BD Biosciences, United States). Cells were immortalized using DDXK-HuBBB^TM^ kit (Eurobio, France) by adapting the manufacturer’s instructions. During the first 10 days of culture, formation of clusters was evaluated by microscopic observation and the cells were stimulated again at day 9 of culture.

**TABLE 1:** Patient’s main characteristics.

	Patient P1	Patient P2	Patient P3

Sex	Female	Male	Male
Age at diagnosis	49 y/o	56 y/o	75 y/o
Age at blood collection	60 y/o	61 y/o	81 y/o
Organ impairment	Kidney, articular and ENT	Pulmonary and ENT	Kidney, pulmonary and neurological
Time since obtaining remission	2 years	2 years	1 year
Treatment	Prednisolone 5 mg/day	Prednisolone 5 mg/day and methotrexate 10 mg/week	Prednisolone 5 mg/day
ANCA type	cANCA	cANCA	cANCA
PR3-ANCA level	23 IU/ml	>177 IU/ml	67 IU/ml
Total B cells CD19+	1/mm^3^	72/mm^3^	36/mm^3^

#### Detection of PR3 Selective IgG by ELISA

After 20 days of expansion, cell culture supernatants were harvested and tested for the presence of IgG by ELISA. The IgG positive wells were selected and retested, and negative clones were eliminated. The presence of anti-PR3 IgG was then tested on positive clones by ELISA using native purified human PR3 (Athens Research, United States) for capture (2 μg/ml) and horseradish peroxidase-labeled goat anti-human IgG (1 μg/ml) for detection. Wells with at least three consecutive positive supernatants in ELISA were amplified to provide enough cells for further testing.

#### Purification of 4C3 Antibody

After confirmation of PR3 specificity, the 4C3 clone was amplified through successive passages from a 24-well plate to a 6-well plate and then into high density flasks (CELLine^TM^, Wheaton^TM^) consisting of two compartments separated by a 10 kDa semi-permeable, cellulose acetate membrane. Monoclonality of 4C3 was confirmed using PCR with IgG heavy chain gene rearrangements (data not shown). Cells and supernatants were harvested every three to 4 days over a period of 70 days. The viability, number of cells and level of production of anti-PR3 IgG were checked during the total culture period. All supernatants collected from cell culture were filtered through a 0.2 μm filter after centrifugation for 10 min at 500 *g* and antibodies contained in supernatants were purified by affinity chromatography (Hitrap^TM^ *Protein A* 1 mL, GE HealthCare) on an AKTA^TM^ device (GE HealthCare, United States). The purity of the different fractions was checked using SDS-PAGE and coloration with Coomassie Blue. The final 4C3 antibody concentration, obtained with a BCA assay, was 5.974 mg/ml. Specificity of 4C3 in the presence of PR3 (1 μg/ml) was confirmed by ELISA.

#### Recombinant Form of 4C3

RNA was isolated from the B cell clone 4C3 and cDNA was synthetized using the Superscript IV first-strand synthesis system (Invitrogen, United States). Sequences corresponding to both the VH and VL regions of the 4C3 clone immunoglobulin were PCR amplified using High-fidelity Taq polymerase Platinum SuperFi II (Invitrogen, United States) and primers were set according to Tiller et al. (34). Briefly, forward primers matched consensus sequences on human κ and γ1 IgG signal regions, reverse primers matched sequences in the constant regions. The PCR products were gel purified (PCR gel extraction kit, Qiagen, Germany) and cloned into pCR4-Blunt-TOPO vector following the manufacturer’s instructions (Invitrogen, United States). DNA from positive clones was purified and analyzed for sequencing. Sequences corresponding to VH and VL domains were PCR amplified, purified and subcloned into pFUSEss-CHIg and pFUSEss-CLIg vectors (Invivogen, France) that enabled the secreted form of both heavy and light IgG chains to be produced. Restriction digests were performed and products ligated using T4 DNA ligase as recommended by the manufacturer. Mammalian HEK-293 cells (ATCC^®^ CRL-1573^TM^) were cultured in Free Style 293F medium (Gibco) at 37°C 8% CO_2_ at 135 rpm. A total of 37.5 μg of plasmid was mixed with 37.5 μl of Free Style Transfection Reagent and incubated for 10 min at room temperature (RT) before addition to cells. Supernatant was collected at day 7 of transfection and r4C3 was purified as for 4C3. The final concentration of r4C3 was 3.5 mg/ml. The specificity of the r4C3 was confirmed by ELISA.

### Characterization of 4C3 and PR3 Interaction

#### Immunofluorescence

2 × 10^5^ purified neutrophils were seeded on a coverslip previously coated with Poly-L-lysine for 20 min at 37°C. After washing with PBS, neutrophils were fixed with either PFA 4% for 10 min or with methanol 100% for 5 min at RT and then permeabilized with PBS-saponin 0.5% for 10 min at RT. Coupling of 4C3 with Alexa Fluor 488 (AF488) was performed with the Protein Labeling Kit (Invitrogen, United States) in accordance with the manufacturer’s instructions. Neutrophils were incubated overnight at 4°C with either 4C3-AF488 (60 μg/ml) or IgG from patient P2 (1/100^*e*^). Rabbit anti-human Fc-AF488 (Life technologies) was used as a secondary antibody for IgG P2 condition. Nuclei from cells were stained with DAPI (4’,6-diamidino-2-phenylindole). Observations were performed using a fluorescence microscope (Nikon Eclipse Ti microscope) and analysis was carried out with ImageJ software.

#### Western Blotting Analysis of PR3 by 4C3

To confirm recognition of native antigen, neutrophils from healthy donors or HeLa cells (ATCC^®^ CCL-2^TM^) were lysed in radioimmunoprecipitation (RIPA) / glycerol buffer (50 mM HEPES, 150 mM NaCl, 10% glycerol, 1% Triton X-100, 2 mM EDTA, and 1% sodium Deoxycholate) supplemented with phosphatase inhibitor (Sigma-Aldrich, United States). Whole-cell lysates, native and reduced PR3 (Athens Research), elastase and cathepsin G (CatG; 5 μg) were separated using SDS-PAGE and transferred to nitrocellulose membranes. Blots were blocked, then incubated with purified 4C3 overnight at 4°C. Hsc70 was used as a reference protein. After staining with horseradish peroxydase-labeled goat anti-human IgG secondary antibody, blots were revealed with an Enhanced Chemiluminescence Detection Kit (GE HealthCare, United States).

#### Flow Cytometry

Purified neutrophils or blood from healthy donors were first incubated with 2.5 μg of human BD Fc block (BD Pharmingen) 10 min at room temperature followed by staining with 4C3-AF488 (2 to 100 μg/ml) or with a commercial murine anti-human PR3 antibody (WGM2-FITC at 10 μg/ml from Hycult Biotech) for 30 min at 4°C before FACS analysis on a MACS Quant (Miltenyi Biotech, Germany). Surface staining with CD45-APC H7/CD3-BV786/CD14-VioBlue/CD15-PE was also performed on blood samples. Data were analyzed with FlowJo software.

#### Affinity Determination of 4C3 to PR3

Affinity measurements of 4C3 and r4C3 for human PR3 were assessed by surface plasmon resonance (SPR) using a T200 apparatus (GE HealthCare). All experiments were performed at 25°C on CM5S dextran sensor chips in PBS flow buffer containing 0.005% of P20 surfactant. 4C3 or r4C3 were captured (200–300 RU) on anti-human IgG Fc-antibody immobilized on CM5S using the anti-human Fc capture kit (GE HealthCare). Increasing concentrations of PR3 protein (Athens Research, Georgia, United States) were injected at 50 μl/min on 4C3 or r4C3 coated flowcells. Phenoxy methane sulphonyl fluoride was added in the injection buffer to minimize the proteinase activity of the PR3. The K_*D*_ were evaluated with a stoichiometric Langmuir fitting model using BiacoreT200 evaluation software.

#### Enzymatic Activity of PR3

Enzymatic activity of PR3 was measured after incubation of 4C3 with 10 nM PR3 at different ratios (10:1, 5:1, and 2:1) for 30 min before adding the fluorescent substrate of PR3 (ABZ-VADnVADYQ-YNO2 from GeneCust) (35). 50 nM α1AT was used to inhibit PR3 activity. The fluorescence was measured through absorbance reading at a wavelength of 420 nm using spectrofluorimeter (SPECTRAmax Gemini^TM^ XPS).

### *In silico* Epitope Mapping of 4C3 on PR3

The 4C3 epitope was predicted using the MAbTope method (36). Briefly, MAbTope is a docking-based method that generates 5 × 10^7^ poses for the antibody-antigen complex and filters these poses in order to obtain the 30 best solutions, using several scoring functions. The interface analysis in these 30 top-ranked solutions allowed identification of residues that exhibit the highest probability of being involved in the interaction. From the prediction, four peptides were designed from the highly probable residues and ordered with an N-terminally-linked biotin (GeneCust, Luxembourg). Each peptide was synthesized in three versions: the actual predicted peptide (numbered.2), and 2 others shifted 3 amino-acids upstream (numbered.1) or downstream (numbered.3). The interaction between the biotinylated peptides and 4C3 or Eculizumab (an anti-C5 mAb), used as a negative control, was assessed using HTRF^®^ (Homogeneous Time Resolved Fluorescence). Briefly, HTRF is a TR-FRET (Time Resolved – Fluorescence Resonance Energy Transfer)-based technology allowing observing protein-protein interaction when these latter ones are coupled to donor (here a rare earth cryptates) and acceptor fluorophores and close enough so that the light emitted by the donor can activate the acceptor (37, 38). Five random peptides were also used as negative controls (CTL1 to 5). All experiments were performed in PPI-Terbium detection buffer (CisBio Bioassays, France). Peptides (2 mM) were first incubated with 8 ng 4C3 for 1 h at RT before adding 5 μl streptavidin-conjugated anti-IgG Fc antibody with HTRF compatible fluorophores, Terbium cryptate and d2, respectively, (CisBio Bioassays, France). 1 h later, the fluorescence emissions at 620 nm and 665 nm were measured on a TriStar^2^ LB 942 Modular microplate reader (Berthold Technologies GmbH & Co. Wildbad, Germany). Data are represented as specific FRET signals calculated as the ratio of the emission at 665 nm divided by the emission at 620 nm subtracted from the binding on the non-relevant Ab (i.e., Eculizumab).

### Functional Characterization of Neutrophil Activation by 4C3

#### Purification of IgG From Serum

For the functional tests, we selected sera of GPA patients with active disease according to clinics, treatment and level of PR3-ANCA. GPA patients with active disease were consecutively diagnosed at the University Hospital Centre of Tours since 2017 to 2019. We purified IgG from five independent GPA patient sera during active phase of the disease after diagnosis (IgG GPA; Table 2) or during remission for patient P2 (IgG P2) and from two independent healthy donors with a protein G Sepharose^TM^ 4 fast flow kit (GE HealthCare, United States). Briefly, non-pooled sera were incubated with protein G for 1 h at RT before elution. Filtration of samples was conducted using a Spin-X^®^ UF Concentrator ultrafiltration system. Purified IgG from non-pooled sera underwent electrophoresis on a 4-12% Bis-tris Gel before staining with Coomassie Blue. The presence of PR3-ANCA in separate IgG preparations from active GPA patients and from patient P2 was confirmed by ELISA using an anti-PR3 EuroImmun kit, as previously described (39), whereas separate purified IgG preparations from healthy donors did not contain any PR3-ANCA.

**TABLE 2:** Main characteristics of active GPA patients, consecutively diagnosed from 2017 to 2019, from which IgG were purified to induce auto-immune activation of neutrophils.

	GPA 1	GPA 2	GPA 3	GPA 4	GPA 5
Sex	Male	Male	Male	Male	Male
Age at diagnosis	68 y/o	71 y/o	79 y/o	77 y/o	53 y/o
Organ impairment	Kidney, neurological and ENT	Articular and ENT	Kidney and articular	Pulmonary and ENT	Kidney and pulmonary
ANCA type	cANCA	cANCA	cANCA	cANCA	cANCA
PR3-ANCA level	83 IU/ml	36 IU/ml	43 IU/ml	112 IU/ml	58 IU/ml

#### Neutrophil Purification and TNFα Priming

Human neutrophils from independent healthy donors were purified using negative magnetic selection with the commercial kit “EasySep Direct Human Neutrophil Isolation Kit” (StemCells, Canada) following the manufacturer’s instructions. At the end of isolation, neutrophils were suspended in HBSS solution without calcium and magnesium. The purity of isolated neutrophils was around >95%, as assessed by flow cytometry (CD15-PE and Live dead, Miltenyi Biotech, Germany). Cells were primed with TNFα (Sigma-Aldrich, United States) at a final concentration of 2 ng/ml for 15 min at 37°C in a water bath (primed-neutrophils) as previously described (13, 40–43).

#### Assessment of ROS Production by Neutrophils

The activation of neutrophils from independent healthy donors was evaluated through ROS production using a dihydrorhodamine 123 (DHR 123) assay as previously described (39, 40, 42). Purified neutrophils were suspended in HBSS with 1 mM Ca^2+^ and 1 mM Mg^2+^ and incubated with 5 μg/ml cytochalasin B (Cayman Chemical, United States) to enhance oxygen radical production, for 5 min at 37°C. Cells were then loaded with 2 μM DHR 123 and 2 mM Sodium Azide (NaN_3_) for 5 min at 37°C under agitation. Primed-neutrophils were incubated with 4C3 (2 to 100 μg/ml), r4C3 or separate IgG preparations from two healthy donors and from four active GPA patients (200 μg/ml) for 45 min at 37°C. A non-relevant antibody (6H4, IgG1κ, anti-ovalbumin) was used as a negative control. A combination of phorbol myristate acetate (PMA, 50 ng/ml) and calcium ionophore (ICa, 10 μM), both powerful neutrophil activators (44, 45), was used as a positive control of neutrophil activation. The reaction was stopped with ice-cold PBS-EDTA (1 mM) before measurement of DHR 123 fluorescence using flow cytometry. For neutralization experiments, pre-treated primed-neutrophils were first incubated with 4C3 (20 μg/ml) for 15 min at 37°C and then separate IgG preparations from five active GPA patients at diagnosis (IgG GPA, 200 μg/ml) were added for 45 additional minutes. To determine the influence of heterogeneous neutrophils from healthy donors, the level of membrane PR3 expression was assessed for each donor using flow cytometry.

#### Degranulation and Adhesion of Neutrophils

Neutrophils were primed with TNFα and stimulated with 4C3 (2 and 20 μg/ml), r4C3 (2 and 20 μg/ml), IgG from GPA patients (200 μg/ml) or PMA-ICa for 45 min at 37°C. After incubation, cells were washed and stained with CD63-FITC (degranulation) or double stained with CD11b VioBlue/CD18 FITC (BD Biosciences, United States; adhesion phenotype) for 20 min at 4°C before analysis using flow cytometry. The percentage of positive cells and MFI were determined using FlowJo software. The degranulation of neutrophils was also assessed by CatG release. Supernatants were harvested and incubated with fluorescent substrate of cathepsin G (ABZ-TPFSGQ-YNO2 from GeneCust) (46) for 30 min before fluorescence reading by spectrofluorimetry at 420 nm. Results are expressed as a ratio of ΔRFU compared to the normalized TNFα condition (basal degranulation).

### Properties of 4C3 Fc Portion

#### Glycosylation Study

The glycoform profile of the 4C3 Fc domain was assessed through high-resolution mass spectrometry using a Vion IMS-QT mass spectrometer hyphenated to an Acquity UPLC H-Class chromatography (Waters, United Kingdom). 800 ng of 4C3 was injected onto an XBridge BEH300 C4 column heated to 90°C. A desalting step was conducted using an isocratic gradient with 95 % solvent A (H2O + 0,1 % formic acid) and 5 % solvent B (acetonitrile + 0,1 % formic acid) for 2 min at 0.5 ml/min. Then, 4C3 was eluted with a flow rate of 0.4 ml/min. Data were acquired on positive mode with an ESI source over a 500 to 4,000 m/z range with a scan rate of 1 Hz and analyzed using UNIFI 1.9.4 software and MaxEnt algorithm for deconvolution (Waters, United Kingdom).

#### Affinity Determination of 4C3 Binding to FcγRIIIB

The interaction between 4C3 and human FcγRIIIB was performed using poly-histidine FcγR (R&D Systems, United States) captured on an immobilized anti-poly-histidine mAb (GE HealthCare, United States). Affinity measurements were assessed using SPR as described above for 4C3 and PR3 and evaluated through a steady-state equilibrium fitting model. Rituximab (an anti-CD20 mAb) was used as a control for FcγRIIIB binding.

### Statistical Analysis

Results are expressed as the means ± SD. Normality of sample distribution was tested prior to conduct any comparison between groups. Non-parametric statistical tests were performed as normality failed, and/or equal variance test failed. Statistical significance was determined using Mann–Whitney rank sum test for unpaired data or using Wilcoxon matched-pairs signed rank test for paired data, depending on the analysis. When necessary, Kruskal Wallis was used to compare groups pairwise. Graphs were produced using GraphPad Prism 7 software (La Jolla, CA, United States).

## Results

### 4C3 Is an IgG1κ PR3-ANCA Able to Recognize Both Soluble PR3 With a High Affinity and Membrane Bound PR3

**FIGURE 1:**
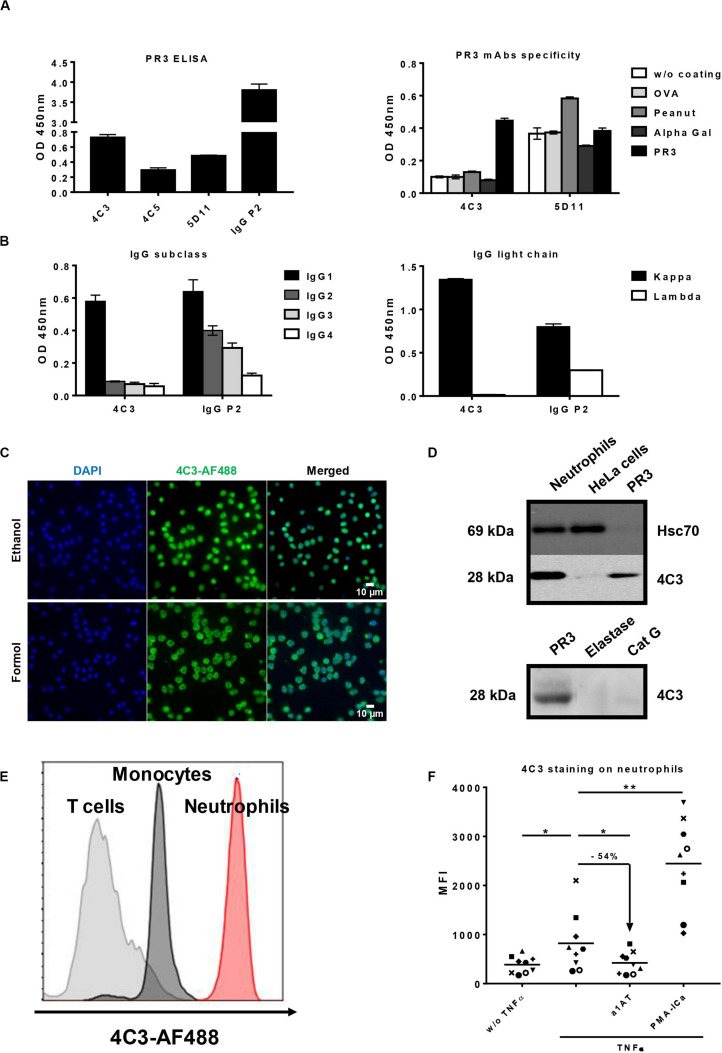
Identification and characterization of an anti-proteinase 3 (PR3) monoclonal human antibody 4C3. **(A)** Identification of a monoclonal human antibody specific to PR3. Binding of native PR3 by ELISA (left panel) in the presence of three antibodies (4C3, 4C5, and 5D11) obtained by immortalization of P2 B cells or serum from P2 containing PR3-ANCA used as a positive control (IgG P2). Similar results were obtained in four independent experiments. Specificity of 4C3 and 5D11 was checked by ELISA (right panel) in the presence of different antigens (PR3, OVA, Peanut, Alpha Gal). *n* = 5. **(B)** 4C3 is an IgG1κ. Subclass (left panel) and nature of light chain (right panel) of 4C3 were determined by ELISA compared to IgG P2. Similar results were obtained in three independent experiments. **(C)** 4C3 has a PR3-ANCA immunofluorescence pattern. Human neutrophils from healthy donors were purified and then fixed with ethanol (upper line) or formol (lower line) before staining with DAPI and 4C3-AF488 (1/100^*e*^). Objective x60. Superposition of fluorescence with ImageJ (Merged). Results from one of three experiments are presented. **(D)** 4C3 specifically recognizes intracellular PR3. Protein lysates (10 μg) from human neutrophils and HeLa cells were loaded before incubation with anti-Hsc70 or 4C3 antibodies for western blotting analysis. PR3 was used as a positive control, elastase and CatG (5 μg) as negative controls. Similar results were obtained in three independent experiments. **(E)** 4C3 has a membrane staining compatible with mbPR3 staining. Blood from a healthy donor was stained with a mixture containing CD45-APC H7/CD3-BV786/CD14-VioBlue/CD15-PE/4C3-AF488. Analysis of 4C3 staining at the surface of T cells (gated on CD3 positive cells, gray histogram), of monocytes (gated on CD14 positive cells, black histogram), and of neutrophils (gated on CD15 positive cells, red histogram). Similar results were obtained in five independent experiments. **(F)** 4C3 binds mbPR3 on quiescent and activated neutrophils. Human purified neutrophils from nine independent healthy donors were primed (TNFα) or not (w/o TNFα) with TNFα at 2 ng/ml for 15 min before staining with 4C3-AF488 (20 μg/ml). Alpha 1 anti-trypsin (α1AT) was used to solubilize mbPR3 by modifying its hydrophobic patch which is involved in its interaction with the membrane. PMA-ICa was used as a positive control of neutrophil activation. Each symbol represents one healthy donor. *n* = 9. NS: Non-significant; *p < 0.05 and **p < 0.005.

Existence of non-pathogenic PR3-ANCA is suggested by the existence of PR3-ANCA in healthy donors (20) and the inconsistent correlation between PR3-ANCA level and disease activity in GPA (15). Moreover, a high level of PR3-ANCA can persist during remission without predicting relapse (16). Therefore, we decided to immortalize B lymphocytes from three GPA patients (P1, P2, and P3) using an EBV-derivative immortalization procedure in order to access the entire B cell repertoire in few weeks and to obtain clones producing fully human Abs. Clinical characteristics of the three GPA patients (P1, P2, and P3) are summarized in Table 1. Blood samples from the GPA patients in remission having a persistent PR3-ANCA level (Table 1) were collected before B cell immortalization but mAbs were successfully obtained for only P2. Indeed, P1 had a too low level of total B lymphocytes and P3 had a high number of granulocytes during culture. P2 was diagnosed with GPA in 2011 following ENT (Ear, Nose and Throat) and pulmonary disorders with alveolar hemorrhage. This patient was first treated with cyclophosphamide for 6 months followed by treatment with corticosteroids and methotrexate. The last biological examination at the time of collection indicated a circulating B-cell count of 72/mm^3^ and a PR3-ANCA level greater than 177 IU/ml. For this patient, we obtained approximatively 1000 wells containing immortalized B lymphocytes and about 50% of these clones produced detectable IgG. From these, three clones (4C3, 4C5, and 5D11) were selected taking into account their capacity to produce antibodies able to recognize PR3 (Figure 1A left panel). Clone 4C5 spontaneously stopped producing Abs. Finally, 4C3 was the only mAb specific to PR3 (Patent *n*°19/11722). Indeed, 4C3 did not recognize non-relevant antigens such as ovalbumin, peanut and alpha-gal, whereas 5D11 did (Figure 1A right panel). Using ELISA, we determined that 4C3 was an IgG1 (Figure 1B left panel) with a light chain kappa (IgG1κ; Figure 1B right panel) whereas the serum from P2 contained different subclasses of IgG. By sequencing, we revealed that 4C3 had a G1m3,1 allotype (data not shown).

To further characterize 4C3 properties, we investigated its ability to bind human neutrophils using IF microscopy. A diffuse cytoplasmic fluorescence was observed, which is the typical cANCA appearance to diagnose GPA patients (Figure 1C). The same staining was observed with the IgG of patient P2 from which 4C3 clone derived confirming the presence of cANCA in this serum (Supplementary Figure 1). To confirm the anti-PR3 specificity, 4C3 was used as the primary antibody in a western blotting analysis performed on protein lysates of primed-neutrophils from healthy donors as well as HeLa cells and human PR3 used as negative and positive controls, respectively. 4C3 recognized human native PR3 with the expected molecular weight (Figure 1D upper panel). By contrast, no staining was observed in HeLa cells, which do not express PR3 (Figure 1D upper panel). In addition, a single band around 28 kDa appeared in the condition of primed neutrophil lysate (Figure 1D upper panel). Finally, we showed that 4C3 did not recognize other neutrophil proteases such as elastase and CatG, which have the same molecular weight as PR3 (Figure 1D lower panel). Taken together, these results show that 4C3 specifically binds PR3.

After demonstrating the recognition of the intracellular neutrophilic PR3, we investigated the ability of 4C3 to bind mbPR3 using flow cytometry of immune cells from human blood. A high level of staining on CD15^+^ neutrophils could be observed. No significant staining could be observed on CD3^+^ T lymphocytes, which do not expose any PR3 and a moderate staining on CD14^+^ monocytes could also be observed. By contrast, a high level of staining on CD15^+^ neutrophils could be observed with a complete right shift of the histogram (Figure 1E). Moreover, we also obtained a bimodal expression of PR3 for two independent healthy donors and no expression of PR3 was found for five healthy donors (data not shown). The observed preferential binding to neutrophils was confirmed through flow cytometry analysis using 4C3 on activated or non-activated purified human neutrophils with different stimuli (Figure 1F). We observed a MFI increase when 4C3 was incubated with TNFα-primed-neutrophils. It should be noted that TNFα has previously been shown to induce translocation of intracellular PR3 to the membrane (47). In contrast, when incubated with α1AT, a natural inhibitor which modifies hydrophobic patch of PR3 and removes the induced mbPR3 from the membrane of primed-neutrophils (29), there was a significant decrease in the MFI. On the other hand, PMA-ICa, strong activators of neutrophils (44, 45), induced a marked increase in the MFI with 4C3 (Figure 1F). Furthermore, this staining was comparable to that obtained with WGM2 mAb, a commercial murine anti-human PR3 antibody, on human neutrophils tested under the same conditions (Supplementary Figure 2). These results confirm that 4C3 is able to bind mbPR3. Finally, using SPR, we showed that 4C3 binds PR3 with a high affinity of 7.4 × 10^–10^ M (Supplementary Table 1). 4C3 from immortalized P2 B cells is a typical IgG1κ PR3-ANCA able to recognize soluble PR3 with a high affinity and mbPR3.

### 4C3 Binds to a New Conformational Epitope on PR3

**FIGURE 2:**
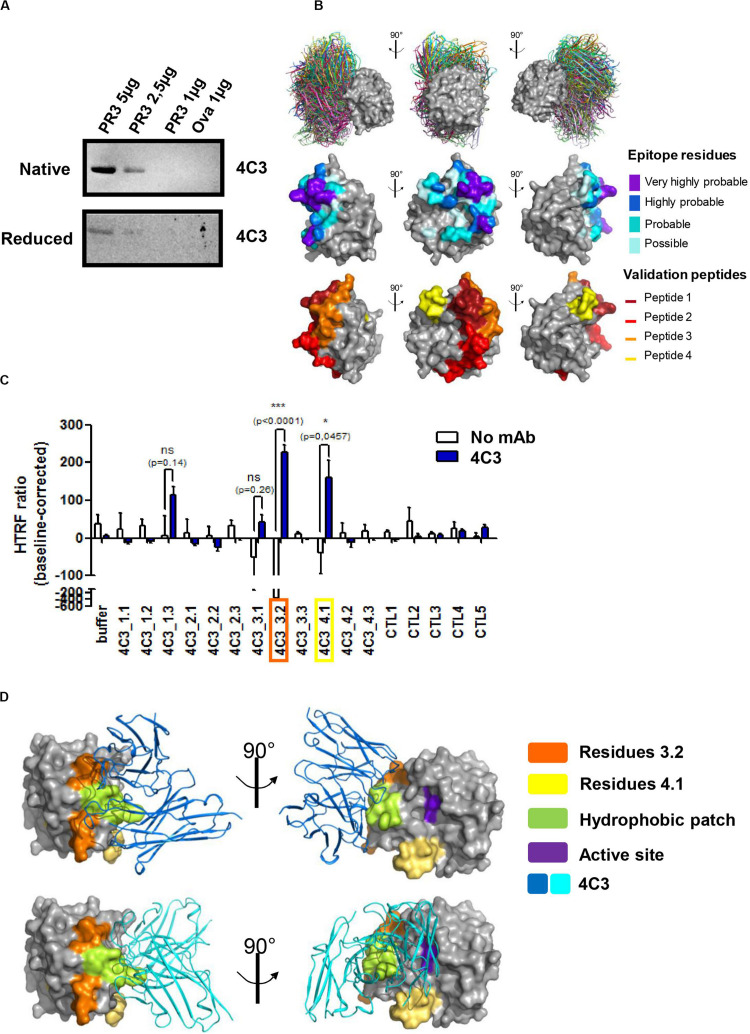
4C3 binds PR3 on a new epitope near the hydrophobic patch and close to the active site. **(A)** 4C3 preferentially recognizes a conformational epitope. Recognition of native (upper gel) or reduced PR3 (lower gel) used at different quantities in western blots after incubation of the membrane with 4C3. Ovalbumin was used as a negative control of binding. **(B)** Prediction of the epitope recognized by 4C3 on PR3. View of the top 30 ranked predicted conformations for the complex between 4C3 and PR3. The target is shown as a gray surface and the antibody in color (first line). The residues that probably belong to the epitope are shown on the structure of PR3 in four categories from purple for the highest probability to light blue for the lowest but still significant probability (second line). Four groups of validation peptides were designed and for each group, the targeted region of 15 residues is presented (third line). **(C)** 4C3 preferentially binds peptides 3.2 and 4.1 of PR3. Analysis by HTRF of the binding of 4C3 with PR3 predicted peptides. Results are expressed as means ± SEM of three independent experiments and each condition was tested in triplicates. Statistical differences are indicated on the graph (*p < 0.05; ***p < 0.0005). **(D)** 4C3 recognizes an epitope near the hydrophobic patch and close to the active site of PR3. The amino acids corresponding to the positive peptides (3.2 in orange and 4.1 in yellow) are indicated on the surface of PR3 (in gray). The catalytic triad (active site) of PR3 is shown in purple while the hydrophobic patch is represented in green. Two possible predicted conformations for the complex 4C3 (in blue) and PR3 (in gray) among the top 30 conformations are presented.

Depending on the patient and the stage of the disease, ANCA recognize conformational rather than sequential epitopes (23, 48). In order to study the role of PR3 conformation in the interaction with 4C3, we performed western blotting analysis using both native and denatured conditions and 4C3 as the primary antibody. PR3 binding was significantly impaired under denatured conditions compared to native conditions, suggesting that 4C3 recognizes a conformational epitope on PR3 (Figure 2A). In order to map such a conformational epitope on PR3, *in silico* docking analyses were performed based on the publically available PR3 structure (49) and structure modeling of VH and VL regions from 4C3 (Patent *n*°19/11722). The 30 top-ranked solutions obtained from the docking procedure for the 4C3-PR3 complex are well grouped on the 3D structure of PR3 and are represented in Figure 2B (Figure 2B upper panel). Amino acid residues of PR3 involved in these top-ranked solutions were divided into four categories according to their raw probability of belonging to the epitope, from purple for the highest probability to light blue for the lowest but still significant probability (Figure 2B middle panel) and on the linear sequence of PR3 (Supplementary Figure 3A). From the predictions of residues belonging to the epitope, four groups of validation peptides, each constituted of 15 residues, were then designed and represented on the 3D structure of PR3 (Figure 2B lower panel). For each of the 4 targeted regions of 15 residues, two more derivative 15-residue long peptides were designed: the first starting and ending 3 residues downstream and the second starting and ending 3 residues upstream (Table 3). Among those peptides, 4C3_3.2 (a.a 152–166) and 4C3_4.1 (a.a 122–136) significantly induced the highest HTRF ratios (Figure 2C) suggesting that these amino acid sequences correspond to the epitope recognized by 4C3 (Supplementary Figure 3A). Therefore, the conformational epitope recognized by 4C3 contains some residues of peptides 3.2 (in orange) and 4.1 (in yellow) exposed on the PR3 surface and located around the hydrophobic patch (in green) and in close vicinity to the active site (in purple) of PR3 as shown on Figure 2D (Figure 2D). Two possible orientations of 4C3 (in blue) among the 30 top-ranked solutions obtained from the docking procedure are also shown in Figure 2D. The first shows that 4C3 masks the hydrophobic patch without preventing access to the active site (Figure 2D, upper panel) while the second represents 4C3 masking the hydrophobic patch with an inaccessible active site (Figure 2D, lower panel). In addition, the binding of 4C3 to PR3 observed using an ELISA was impaired by 44% in the presence of α1AT, inducing a conformational change of PR3 and preventing the binding of the PR3-ANCA-recognizing epitope 1 to PR3 (29) (Supplementary Figure 3B). This confirms that the epitope recognized by 4C3 is partially masked by α1AT and that some amino acid residues involved in this epitope are localized in the epitope 1, close to the active site of PR3. Moreover, we demonstrated that incubation of PR3 with molar excess of 4C3 did not result in a decrease in PR3 enzymatic activity, unlike with α1AT, which induced an inhibition (Supplementary Figure 3C), thus suggesting that the active site is still accessible in the presence of 4C3.

**TABLE 3:** Validation peptide sequences.

Peptide	Start	Sequence	End
4C3_1.1	188	GIDSFVIWGCATRLF	202
4C3_1.2	191	SFVIWGCATRLFPDF	205
4C3_1.3	194	IWGCATRLFPDFFTR	208
4C3_2.1	72	HFSVAQVFLNNYDAE	86
4C3_2.2	75	VAQVFLNNYDAENKL	89
4C3_2.3	78	VFLNNYDAENKLNDI	92
4C3_3.1	149	TVVTFFCRPHNICTF	163
4C3_3.2	152	TFFCRPHNICTFVPR	166
4C3_3.3	155	CRPHNICTFVPRRKA	169
4C3_4.1	122	GTQCLAMGWGRVGAH	136
4C3_4.2	125	CLAMGWGRVGAHDPP	139
4C3_4.3	128	GWGRVGAHDPPAQV	142

To conclude, 4C3 recognizes a conformational epitope on PR3 localized close to the active site and the hydrophobic patch on a region overlapping that of epitope 1 without affecting PR3 enzymatic activity.

### 4C3 Inhibits Neutrophil Activation Induced by Polyclonal PR3-ANCA

Proteinase 3-ANCA have a central role in GPA, by cross-linking mbPR3 and Fc gamma receptors at the surface of neutrophils, causing auto-immune activation of the latter (7, 8). *In vitro* stimulation of TNFα-primed-neutrophils by chimeric anti-human PR3 mAbs or purified IgG from GPA patients (IgG GPA) induces their auto-immune activation leading to ROS production, protease release by degranulation and adhesion molecules upregulation (10, 12, 13).

**FIGURE 3:**
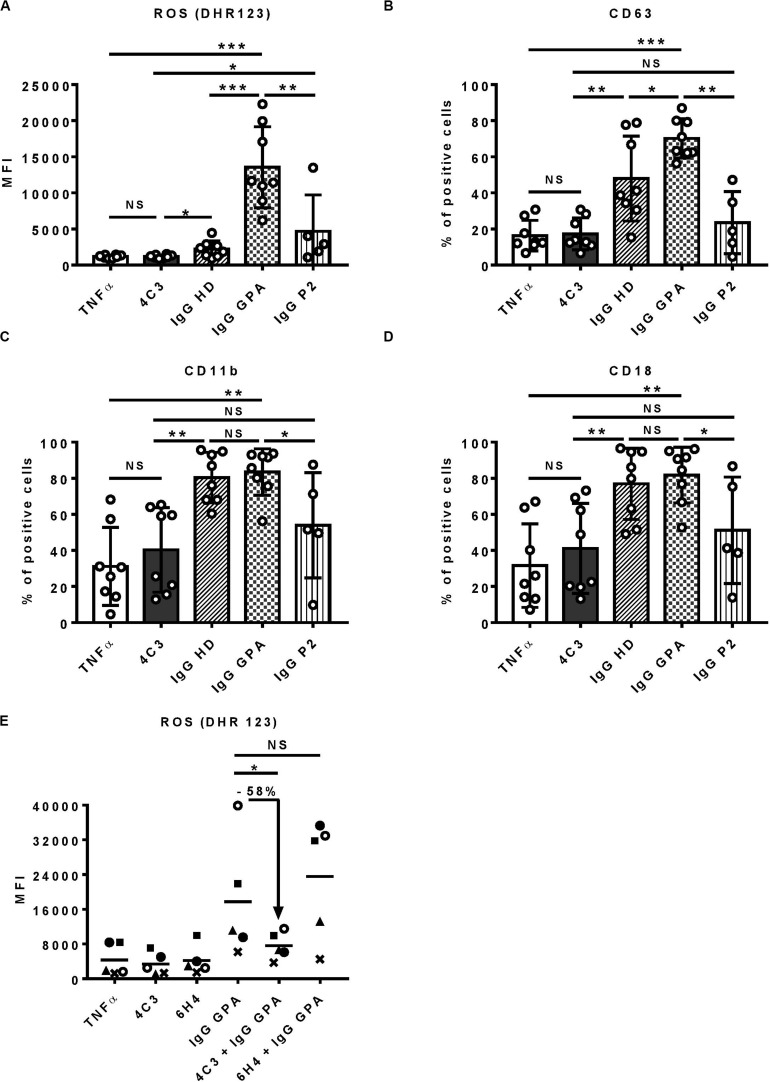
4C3 does not allow activation of human neutrophils and inhibits PR3-ANCA-induced activation. Purified neutrophils from eight independent healthy donors were primed with TNFα (2 ng/ml) for 15 min at 37°C (white columns) before incubation for 45 min with 4C3 (gray columns), separate (non-pooled) IgG preparations from two healthy donors (hatched columns) or from four active GPA patients at diagnosis (checkered columns) or IgG from patient P2 (vertical lines). **(A)** 4C3 does not induce production of reactive oxygen species (ROS) by neutrophils. ROS production was assessed by measuring the fluorescence (MFI) of DHR 123 by flow cytometry. **(B)** 4C3 does not induce degranulation. The degranulation of neutrophils was assessed by CD63 expression represented in percentage of positive cells. **(C, D)** 4C3 does not increase the adhesion phenotype of neutrophils. The adhesion criteria of neutrophils was assessed by measuring CD11b **(C)** and CD18 **(D)** surface expressions by flow cytometry. Results are expressed as mean ± SEM obtained in eight independent experiments with a circle representing one experiment. NS: Non-significant; *p < 0.05; **p < 0.005; and ***p < 0.0005. **(E)** 4C3 is able to inhibit ROS production induced by polyclonal PR3-ANCA. After priming, neutrophils from five independent healthy donors were first incubated for 45 min with 4C3 (gray column) or 6H4 (non-relevant mAb obtained under the same conditions as 4C3; black column) before addition of separate IgG preparations purified from five independent active GPA patients (IgG GPA at 200 μg/ml). Each symbols represents one healthy donor. ROS production was assessed by measuring the fluorescence (MFI) of DHR 123 by flow cytometry. The percentage of inhibition induced by 4C3 in ROS production is indicated on the graph. NS: Non-significant; *p < 0.05. *n* = 5.

To characterize 4C3 further, we investigated the *in vitro* functionality of 4C3 on human primed-neutrophils (Figure 3). First, we assessed ROS production induced by primed-neutrophils after 4C3 stimulation. 4C3 was not able to induce ROS production whereas separate IgG preparations from active GPA patients at diagnosis (IgG GPA) led to marked production. On the contrary, separate IgG preparations from healthy donors (IgG HD), obtained and used under the same conditions, did not allow ROS production by primed-neutrophils (Figure 3A). Interestingly, neutrophil stimulation by non-pooled purified IgG from the patient P2 did not induce significant ROS production compared to IgG GPA purified from GPA patients at diagnosis (Figure 3A). We checked that neutrophil activation induced by purified IgG GPA (from non-pooled preparations) was specific to PR3 activation as pre-incubating neutrophils with α1AT strongly decreased ROS production induced by IgG GPA (data not shown). Primed-neutrophil stimulation with lower (2 μg/ml) and higher (100 μg/ml) concentrations of 4C3 showed no increase in ROS production (Supplementary Figure 4A). The capacity of neutrophils to degranulate was measured by CD63 expression at the neutrophil surface and also by proteinase release in supernatants after activation. Similar results were obtained with no difference between 4C3 stimulation and TNFα condition or IgG from patient P2 on CD63 staining and CatG activity (Figure 3B and Supplementary Figure 4B). Moreover, we analyzed the expression of CD11b and CD18 (Mac1 complex) to explore the adhesion phenotype of neutrophils following 4C3 stimulation. The presence of 4C3 did not induce any increased surface expression of CD11b/CD18 whereas non-pooled IgG preparations from healthy donors or GPA patients at diagnosis significantly induced upregulation of these two adhesion markers (Figures 3C,D). It should be noted that IgG from patient P2 induced an intermediate adhesion phenotype (Figures 3C,D).

We then studied the capacity of 4C3 to inhibit neutrophil activation induced by polyclonal PR3-ANCA. Neutrophils were pre-incubated with 4C3 or with 6H4, a human anti-ovalbumin IgG1κ strictly produced and stored under the same conditions as 4C3. Purified IgG was obtained from five distinct GPA patients at diagnosis (IgG GPA) and then added to the two neutrophil preparations and ROS production analyzed as previously described. Interestingly, 4C3 was able to reduce the ROS production induced by IgG GPA stimulation with an inhibition mean of 58% on the five different patients (percentages of inhibition from 36 to 71%; Figure 3E). On the contrary, 6H4, a non-relevant antibody, was not able to inhibit the effect of IgG GPA (Figure 3E). Taken together, these results indicate that 4C3 did not activate neutrophils and was able to inhibit neutrophil activation induced by IgG GPA.

### Properties of 4C3 Fc Portion do Not Explain the Non-activation of Neutrophils

We previously demonstrated that 4C3 was not able to induce primed-neutrophil activation. Therefore, we studied the properties of its Fc portion in order to investigate whether this unexpected feature could result from a defect in the 4C3 Fc portion. 4C3 was able to bind FcγRIIIB (CD16b) with an affinity of 2.7 × 10^–6^ M (Supplementary Figure 5) and prevented the binding of an anti-FcγRIIA (anti-CD32), similarly to rituximab used as a control, on the THP-1 human monocytic cell line (data not shown). These results suggest that 4C3 is able to bind the two mains Fc gamma receptors at the neutrophil surface.

**FIGURE 4:**
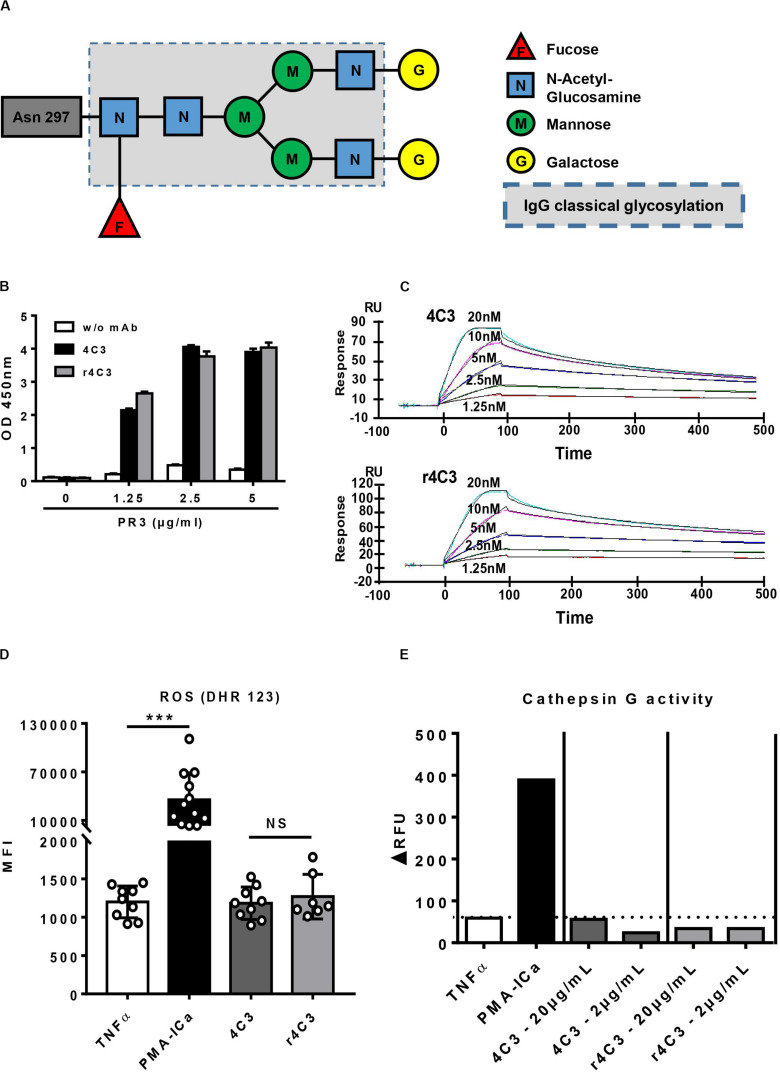
Non-activation of neutrophils by 4C3 is independent of a defect in the Fc portion. **(A)** 4C3 has a G2F profile of glycosylation with an increase in galactose residues. Glycosylation of 4C3 in asparagine 297, obtained by mass spectrometry, is presented compared to classical glycosylation of IgG (dotted line). **(B)** The recombinant form of 4C3 (r4C3) has the same ability to bind PR3 as native 4C3. Binding of 4C3 (black columns) and recombinant 4C3 (r4C3; gray columns) to PR3 by ELISA. BSA (0 μg of PR3) was used as a negative control of binding. Results are expressed in Optical Density. The results of one experiment out of three are presented. **(C)** r4C3 has an affinity for PR3 comparable to that of 4C3. Increasing concentrations of PR3 (1.25 to 20 nM) were injected on 4C3 and r4C3 coated flow cells for 500 min. Binding was monitored as an increase in SPR signal expressed in resonance units (RU). The results of one experiment out of three are presented. **(D)** r4C3 does not induce ROS production by neutrophils. Purified neutrophils from nine independent healthy donors were primed with TNFα (2 ng/ml) for 15 min at 37°C (white histogram) before incubation for 45 min with PMA-ICa (black column), 4C3 (dark gray column) or r4C3 (light gray column). ROS production was assessed by measuring the fluorescence (MFI) of DHR 123 by flow cytometry. *n* = 9. NS: Non-significant. ***p < 0.0005. **(E)** r4C3 does not increase cathepsin G activity. Purified neutrophils from three independent healthy donors were primed with TNFα (2 ng/ml; white histogram) for 15 min at 37°C before incubation for 45 min with 4C3 (dark gray columns) or r4C3 (light gray columns) at 2 and 20 μg/ml or PMA-ICa (black column). Cathepsin G activity was assessed after adding the substrate to the supernatants of neutrophils and reading the fluorescence (ΔRFU) by spectrofluorimetry. The results of one experiment out of three are presented.

Moreover, mass-spectrometry profiling of the glycosylation pattern of 4C3 demonstrated an increase in galactose residues revealing a G2F profile compared to the classical asparagine 297-linked glycosylation of an IgG1 (Figure 4A).

In order to confirm that the non-activation of primed-neutrophils induced by 4C3 was not due to the properties of its Fc portion, we engineered a recombinant form of 4C3 (r4C3) with a different Fc portion. After 4C3 sequencing, an IgG1 recombinant form was produced with a different allotype (G1m17,1) in a different cell culture system (HEK). Therefore, 4C3 and r4C3 had the same variable heavy and light chain region but a slightly different Fc portion. We first showed that 4C3 and r4C3 had the same ability to recognize PR3 using ELISA (Figure 4B). This result was confirmed by SPR as r4C3 had a similar affinity for PR3 to 4C3 (Figure 4C and Supplementary Table 1). Finally, primed-neutrophil stimulation with r4C3 did not cause significant ROS production as the negative control and 4C3 contrary to the powerful activator PMA-ICa (Figure 4D) and no CatG release was observed in the presence of r4C3 (Figure 4E).

To conclude, 4C3 does not induce primed-neutrophils activation despite having a functional Fc portion. r4C3, with a different but functional Fc portion, does not induce primed-neutrophils activation either. These results suggest that non-activation of primed-neutrophils by 4C3 is not due to the Fc portion properties of 4C3, but rather to its Fab domain and therefore to the epitope recognized on PR3.

## Discussion

Clinical observations suggest the existence of non-pathogenic PR3-ANCA in GPA: 1/correlation between PR3-ANCA level and disease activity is inconsistent (15), 2/a high level of PR3-ANCA can persist during remission without predicting relapse (16), and 3/PR3-ANCA can be detected in healthy donors (20). Furthermore, the existence of non-pathogenic PR3-ANCA could be rational to scavenge neutrophils debris. Indeed, a negative correlation between circulating PR3 and ANCA in AAV patients in remission has been observed and is an indirect argument supporting this hypothesis (50). To our knowledge the existence of non-pathogenic PR3-ANCA has never been demonstrated. Indeed, PR3-ANCA pathogenicity, i.e., their potential to induce auto-immune activation of neutrophils, is influenced by several factors including the level of mbPR3 on neutrophils (9), their epitope on PR3 (23), their type of Ig (14, 30, 51) and their glycosylation (31–33). All these characteristics have been studied with chimeric or murine anti-human PR3 antibodies or with human anti-PR3 polyclonal IgG but never with a human monoclonal anti-PR3 antibody.

In this study, we obtained and sequenced for the first time a fully human PR3-ANCA mAb (4C3) by immortalization of B lymphocytes from a GPA patient in remission who had a persistently high level of PR3-ANCA. Most of monoclonal antibodies are produced by transfection of genes encoding human immunoglobulin from cell lines or hybridomas after immunization. These antibodies are not fully human. In this study, we chose B cell immortalization, starting directly from human B lymphocytes, in order to obtain a fully human mAb closer to those produced in GPA patients. The main interests with this method are that the fully human monoclonal antibodies obtained had relevant PR3 recognition, Fc functionality and glycosylation similar or near to that of the PR3-ANCA from P2 patient (a patient in remission).

4C3 has all the characteristics of a classic PR3-ANCA: a cANCA IF pattern and a specificity toward PR3 (1). Furthermore, 4C3 is an IgG1κ, the most frequent subclass of PR3-ANCA able to induce neutrophil activation (14, 30, 41). Nevertheless, 4C3 appears to be non-pathogenic as it was not able to induce *in vitro* human neutrophil activation contrary to human polyclonal PR3-ANCA from the acute phase of GPA. A limitation of this presented study is the investigation of only one mAb anti-PR3 without any activating mAb obtained from a GPA patient at diagnosis. However, the existence of non-pathogenic PR3-ANCA, as 4C3, could explain why PR3-ANCA can persist during remission without predicting relapse and why they can be found in healthy donors.

Using the original MAbTope method and HTRF assay, we were able to demonstrate that 4C3 recognized a conformational epitope on PR3 and amino acid residues involved in this epitope are contained in linear peptides 4C3_3.2 (a.a 152–166) and 4C3_4.1 (a.a 122–136). Based on a 3D structure, the conformational epitope recognized by 4C3 is localized around the hydrophobic patch that allows the PR3 to be anchored to the neutrophil membrane (29). However, this particular location did not prevent 4C3 binding to the neutrophil surface. Indeed, some commercial antibodies targeting epitope 5, situated in the hydrophobic patch region, are not able to bind mbPR3 (29). Moreover, α1AT complexation with PR3 partially inhibits 4C3 binding on its epitope suggesting that some amino acid residues involved in this epitope are localized on epitope 1. Indeed, the binding of PR3-ANCA recognizing epitope 1 is impaired by α1AT inducing a conformational modification of PR3 (29). The main epitope region recognized by 4C3 could be confirmed by inhibition ELISA experiments using murine anti-PR3 monoclonal antibodies as previously performed with patient sera containing PR3-ANCA (52). We also confirmed the binding of 4C3 to mbPR3 on monocytes and neutrophils compared to a commercial murine anti-human PR3 mAb recognizing epitope 3 on PR3 (25). The capacity of 4C3 to bind mbPR3 after the priming of neutrophils was a necessary prerequisite to study neutrophil activation (9). Finally, it should be noted that it is the first time that the affinity of a human PR3-ANCA to PR3 has been evaluated. 4C3 binds PR3 with a high affinity (K_*D*_: 7.4 × 10^–10^ M). This affinity is a thousand-fold higher than that of IgG to FcγR (53) and supports that the binding of PR3-ANCA to mbPR3 is a fundamental step in fixing PR3-ANCA to the neutrophil surface.

Despite being a human IgG1 PR3-ANCA with high binding affinity to mbPR3, 4C3 was not able to induce the activation of primed-neutrophils. Indeed, primed-neutrophil stimulation with 4C3 did not induce any ROS production, degranulation or CD11b/CD18 upregulation by human neutrophils. Furthermore, neutrophil pre-incubation with 4C3 significantly reduced by 58% the ROS production induced by pathogenic IgG GPA stimulation. GPA is still a serious disease despite current therapies used (54), with a mortality rate of 25% at 5 years (3), significant morbidity related to the disease and its management (55) and a high risk of relapse (56). Thus, 4C3 ability to inhibit the fundamental implication of PR3-ANCA binding to PR3 in the autoimmune activation of neutrophils seems an attractive way to develop new therapies. This result is in line with results obtained with α1AT, a serine protease inhibitor, which clears PR3 from the membrane and thus prevents its binding to PR3-ANCA. Indeed, it has already been demonstrated that pre-incubating neutrophil with α1AT decreases ROS production induced by PR3-ANCA (40, 57). In our study, 4C3 did not clear mbPR3, as α1AT did, but may act in competition with pathogenic PR3-ANCA. Different hypotheses could be raised: the first suggests that the interaction of 4C3 with PR3 changes the conformation of the latter and consequently changes the major epitope of pathogenic PR3-ANCA. The second and third possibilities are that when 4C3 binds mbPR3, the major epitope of pathogenic PR3-ANCA is masked or 4C3 Fc domain limits their binding to FcγRs. In all cases, this prevents the interaction of pathogenic PR3-ANCA with neutrophils. Therefore, the 4C3 capacity to inhibit auto-immune activation of neutrophils induced by polyclonal IgG PR3-ANCA, although tested by neutrophil pre-incubation with 4C3, could be a promising therapeutic strategy at the active phase of the disease. Indeed, 4C3 has the advantage to have a high-affinity to PR3 and has a long half-life of an IgG1. 4C3 could compete on neutrophils fixation, especially on young neutrophils, before pathogenic PR3-ANCA binding. However, the non-pathogenic characteristic of 4C3 needs further investigation but is an interesting avenue to develop regarding the pathogenicity of PR3-ANCA.

The non-activation of neutrophils by 4C3 could not be explained by the nature of the mAb because chimeric anti-human PR3 mAb was able to induce neutrophil activation in the literature (14, 58). Furthermore, polyclonal stimulation of neutrophils with purified IgG from remission patient P2 (from which 4C3 was obtained and containing a high PR3-ANCA level), did not cause significant neutrophil activation compared to IgG GPA. The correlation between neutrophils activation induced by IgG fractions from GPA patients and disease activity of these patients has already been observed (51). This interesting result highlights that a polyclonal solution of PR3-ANCA is not sufficient to induce neutrophil activation probably because the proportion of pathogenic and non-pathogenic PR3-ANCA is variable from one patient to another. We can therefore deduce that the original patient P2 had non-pathogenic PR3-ANCA. Finally, it must be underlined that auto-immune activation of neutrophils by IgG ANCA was recently challenged by Popat and Robson. Indeed, purified IgG from AAV patient sera, even in active disease, did not induce neutrophil activation from two different healthy donors (59). Interestingly, the same IgG preparations obtained from MPO-ANCA positive patients but not from PR3-ANCA positive patients were used by the same group, in another study, and promoted inflammation through monocyte stimulation (60). Another study suggested that neutrophil activation induced by IgG ANCA, mostly observed in the literature, could be related to the persistence, after purification, of elements from patient sera and not to IgG ANCA (61). Regardless, in our study, the purity of IgG was verified by SDS-PAGE and only one band was visible.

To investigate further the non-pathogenic characteristic of 4C3, we studied its Fc portion properties and developed a recombinant form to eliminate the presence of a defect in the Fc portion of 4C3. We demonstrated that the 4C3 Fc domain was functional with a capacity to bind both FcγRIIA and FcγRIIIB with an expected affinity for an IgG1 (53). FcγRIIA and FcγRIIIB, the two mains FcγR present at the neutrophil surface (62), are involved in ROS production, NETosis and degranulation induced by PR3-ANCA stimulation (7, 9). Furthermore, deglycosylation of the PR3-ANCA Fc portion attenuates neutrophil activation (63), but 4C3 has a glycosylated Fc portion. However, 4C3 glycosylation is with an elevated level of galactose residues, i.e., a G2F profile. Modifications in glycosylation of IgG linked with asparagine 297 are known to be involved in autoimmune diseases (31). IgG from GPA patients show low levels of bisection, sialylation and galactosylation in the active phase of the disease (32, 33, 64, 65) and, in one study, clinical remission was associated with complete glycan normalization of total IgG1 (33). Moreover, hyposialylation of IgG PR3-ANCA is correlated with disease activity and with ROS production by neutrophils (65). Contrary to IgG found in the active phase of the disease with low levels of galactosylation, 4C3 has an elevated level of galactose residues. This “anti-inflammatory” profile could also explain its non-pathogenic characteristic.

Results obtained with the recombinant form of 4C3 (r4C3) are in agreement with the fact that the non-activation property of 4C3 could not be due to a defect in the Fc portion. r4C3 has a different allotype, G1m17,1, which is one of the main allelic form of IgG1 (66) and is functional as it is classically used in therapy, e.g., rituximab (67). Therefore, the non-activation of neutrophils by 4C3 is not due to a particularity of its Fc portion because r4C3 with a functional Fc portion did not induce neutrophil activation either. Furthermore, we can hypothesize that IgG1 allotype is not implicated in neutrophil activation by PR3-ANCA as it has been suggested for other immune mechanisms (68).

The non-pathogenic character of 4C3 seems to be related to its epitope targeted on PR3. In the literature, ANCA mainly recognize conformational epitopes (23, 48) with variations between patients and in the same patient during the course of the disease according to disease activity (22, 69). PR3-ANCA found in the active phase of the disease have been shown mainly to recognize a region close to the active site, called epitope 1 (23–25), and to inhibit *in vitro* PR3 enzymatic activity (26–28). Based on the linear representation of known PR3 epitopes in GPA from van der Geld et al. (23), we can conclude that amino acid residues corresponding to the conformational epitope recognized by 4C3 are localized on the C-terminal region in the sequence of the complementary peptide of PR3 (a.a 87–172) which is hypothesized to initiate autoimmunity through idiotype–anti-idiotype response (23). This new epitope is different from any epitope already described in the literature (23) and 4C3 binding on this epitope does not affect *in vitro* PR3 enzymatic activity. This last result strengthens the non-pathogenic character of 4C3 because, in contrast, pathogenic PR3-ANCA found in the active phase of the disease generally inhibit *in vitro* PR3 enzymatic activity (26–28). The non-pathogenic characteristic of 4C3 could be explained by its binding to the particular epitope of PR3 and by a particular Fc domain orientation preventing its binding to FcγR. Several epitopes are associated with PR3-ANCA but, unlike MPO-ANCA, none is specifically associated with the active phase of the disease or remission (23, 48). Indeed, Roth et al. have described MPO-ANCA, directed against a linear epitope on MPO (AA 447–459), exclusive to disease activity (48). The level of those MPO-ANCA correlates extremely well with disease activity whereas the total MPO-ANCA level did not (48). Furthermore, epitopes of asymptomatic or natural auto-antibodies were localized close to epitopes related to active disease (48). The hypothesis of discovering a specific epitope associated with remission in PR3-AAV, e.g., the one targeted by 4C3, seems relevant as specific epitopes associated with active disease or remission in MPO-AAV have been already described. As shown for MPO, the epitope recognized by 4C3 is close to epitopes recognized by pathogenic PR3-ANCA, i.e., close to the active site and to the epitope 1 region.

To conclude, non-pathogenic PR3-ANCA, which are not able to activate neutrophils, exist and can be isolated in GPA patients in remission. This could explain why PR3-ANCA can persist during remission without predicting relapse and can also be found in healthy donors. The non-pathogenic characteristic of 4C3 could be related to its ability to recognize a particular conformational epitope on PR3. This newly described epitope could be linked to remission and might be used as a remission biomarker within the framework of “personalized medicine approach” in PR3-AAV management suggested by Osman et al. (70). Improved understanding of differences between non-pathogenic and pathogenic PR3-ANCA is necessary to develop these new biomarkers in GPA. Finally, our human mAb 4C3, which has an inhibitory property, could be interesting in a therapy to inhibit auto-immune activation of neutrophils induced by PR3-ANCA of GPA patients in the active phase of the disease.

## Data Availability Statement

The datasets presented in this study can be found in online repositories. The names of the repository/repositories and accession number(s) can be found at: http://www.wwpdb.org/, PDB:4ODX, http://www.wwpdb.org/, PDB:3SKJ, and http://www.wwpdb.org/, PDB:1FUJ.

## Ethics Statement

Written informed consent was obtained from the individuals for the publication of any potentially identifiable images or data included in this article.

## Author Contributions

CH and BK conceived the study. RL, CH, and JG conceived and designed the experiments while JG, EM, YG, JM, MD, SS, and RL performed them. JG and RL were involved in data analysis. MP performed SPR experiments. AP and AM did epitope mapping. JG, RL, BK, HW, DN, and CH wrote the manuscript. All the authors have read and approved the revised manuscript.

## Conflict of Interest

AM was employed by MAbSilico SAS. The remaining authors declare that the research was conducted in the absence of any commercial or financial relationships that could be construed as a potential conflict of interest.
