# Supplementary material for: 4C3 Human Monoclonal Antibody: A Proof of Concept for Non-pathogenic Proteinase 3 Anti-neutrophil Cytoplasmic Antibodies in Granulomatosis With Polyangiitis
Source: Front Immunol. 2020 Sep 25;11:573040. doi: 10.3389/fimmu.2020.573040 (PMC7546423; doi:10.3389/fimmu.2020.573040)
Supplement: Supplementary file 1 [file Data_Sheet_1.pdf]

Supplementary Figure 1

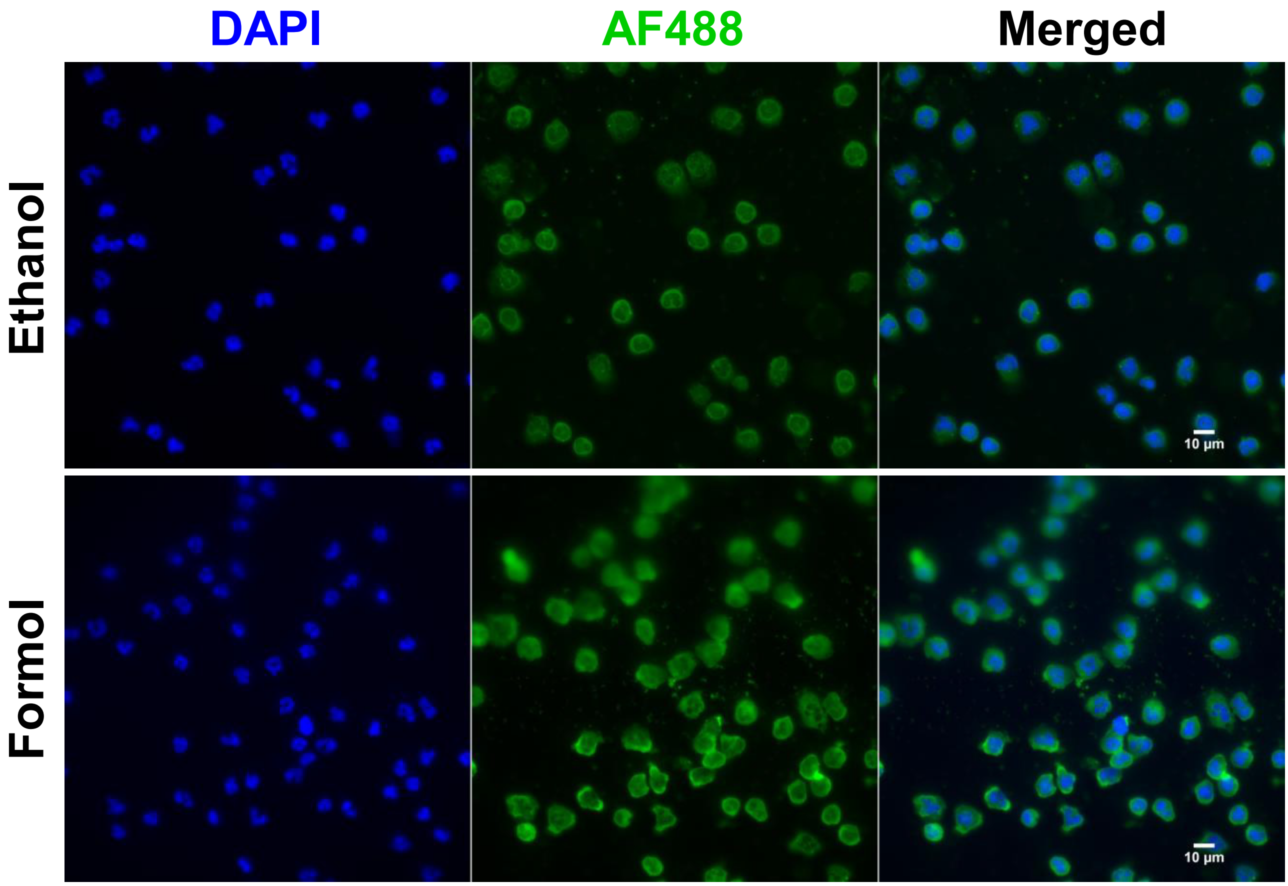

Supplementary figure 1: Serum from patient P2 contains PR3-ANCA. Human purified neutrophils from healthy donor were purified and then fixed with ethanol (upper line) or formol (lower line) before incubation with IgG from patient P2 (1/100^{e}). Staining with DAPI and rabbit anti-human AF488. Objective x60. Superposition of fluorescence with ImageJ (Merged). Results from one of three experiments are presented.

Supplementary Figure 2

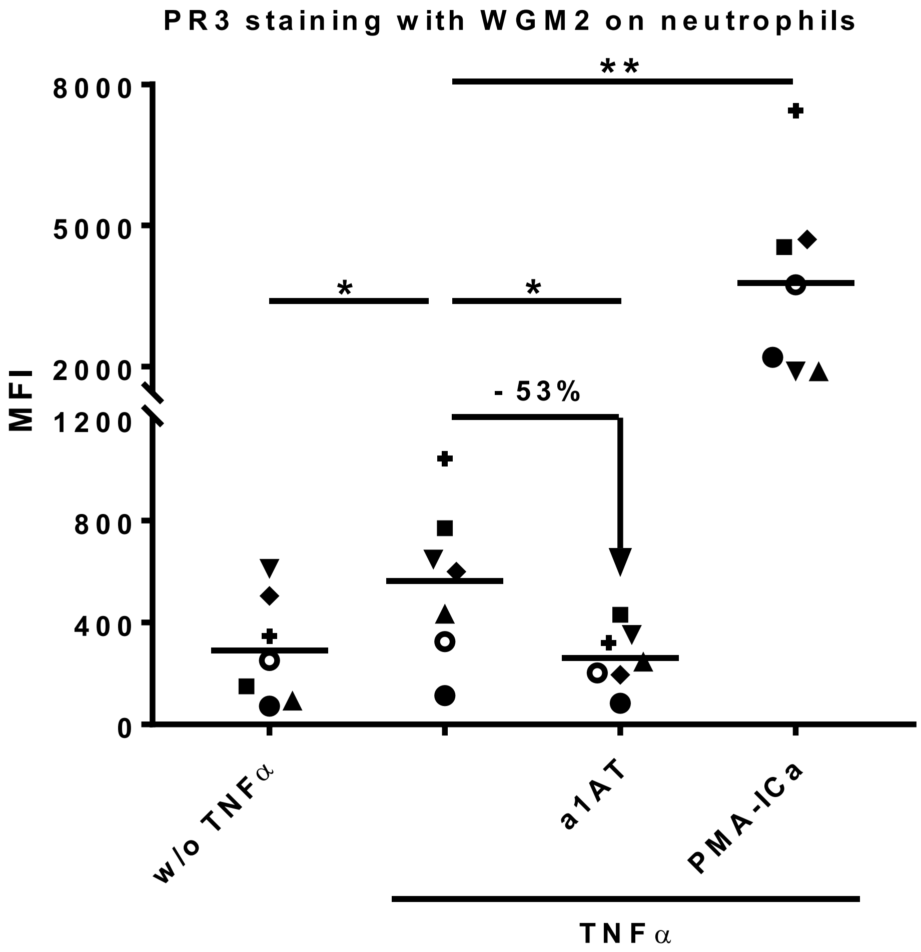

Supplementary figure 2: PR3 staining with WGM2 antibody on human neutrophils. Human purified neutrophils from seven independent healthy donors were primed (TNFα) or not (w/o TNFα) with TNFα at 2 ng / ml for 15 minutes before staining with WGM2-FITC (10 µg / ml). Alpha 1 anti-trypsin (a1AT) was used to solubilize mbPR3 by modifying its hydrophobic patch which is involved in its interaction with the membrane. PMA-ICa was used as a positive control of neutrophil activation. Each symbol represents one healthy donor. n=7. NS: Non-significant ; **$p<0.05$; **$p<0.005$.*

Supplementary Figure 3

A

```
  1 IVGGHEAQPH SRPYMASLQM RGNPGSHFCG GTLIHPSFVL TAAHCLRDIP
 51 QRLVNVVLGA HNVRTQEPTQ QHFSVAQVFL NNYDAENKLN DILLIQLSSP
101 ANLSASVATV QLPQQDQPVP HGTQCLAMGW GRVGAHDPPA QVLQELNVTV
151 VTFFCRPHNI CTFVPRRKAG ICFGDSGGPL ICDGIIQGID SFVIWGCATR
201 LFPDFFTRVA LYVDWIRSTL R
```

Hydrophobic patch   Catalytic triad (active site)

Validation peptides
- Peptide 3
- Peptide 4

Epitope residues
- Very highly probable
- Highly probable
- Probable
- Possible

B

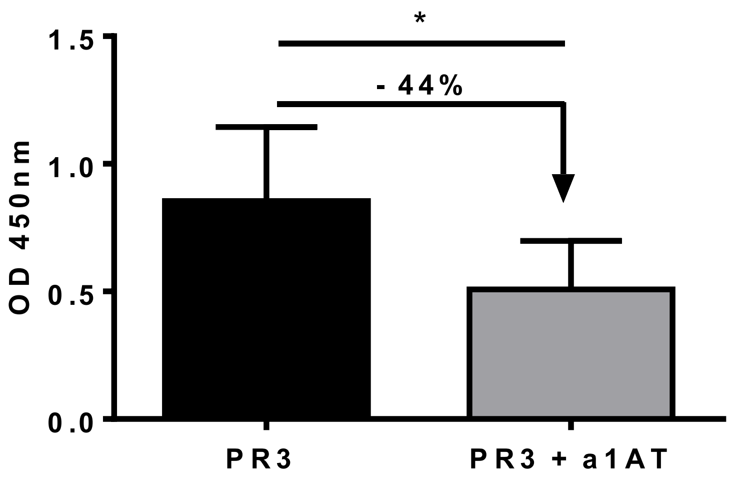

C

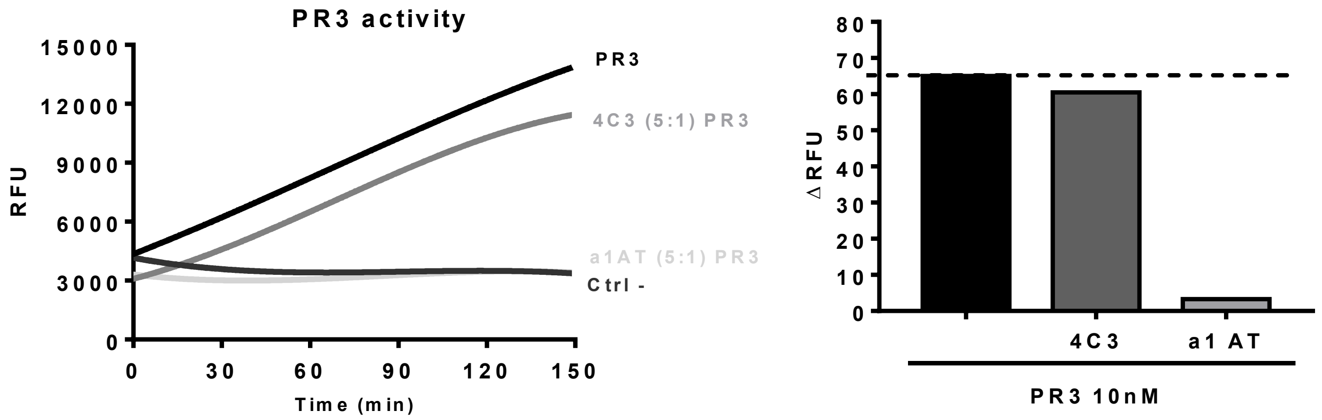

Supplementary figure 3: 4C3 targets an epitope close to the hydrophobic patch and active site, which does not prevent the enzymatic activity of PR3

(A) Residues predicted to belong to the epitope and validation peptides are shown on PR3 linear sequence. Amino acids of the hydrophobic patch are circled in grey and those of active site (catalytic triad) are circled in blue. **(B) Alpha 1 anti-trypsin partially inhibits the binding of 4C3 on PR3.** PR3 (2 µg / ml) was incubated (PR3 + a1AT) or not (PR3) with a1AT at a ratio of 1 PR3 per 5 a1AT for 1 hour at 37°C before coating. Results are expressed as mean ± SEM of optical density obtained in eight independent experiments. The percentage of inhibition of PR3 binding by 4C3 is indicated on the graph. **p<0.05*. **(C) 4C3 does not inhibit the enzymatic activity of PR3.** Native PR3 (10 nM) was incubated or not with 4C3 or a1AT (50 nM) for 30 minutes before adding fluorescent substrate of PR3. PR3 activity was measured for 160 minutes using spectrofluorimetry. The negative control (Ctrl-) corresponds to the substrate alone. Right panel represents the difference of PR3 activity with 4C3 and a1AT expressed in ΔRFU obtained at the ratio of 5 4C3 for 1 PR3. Similar results were obtained at ratios 10 :1 and 2 :1. Results of one of five experiments are presented.

Supplementary Figure 4

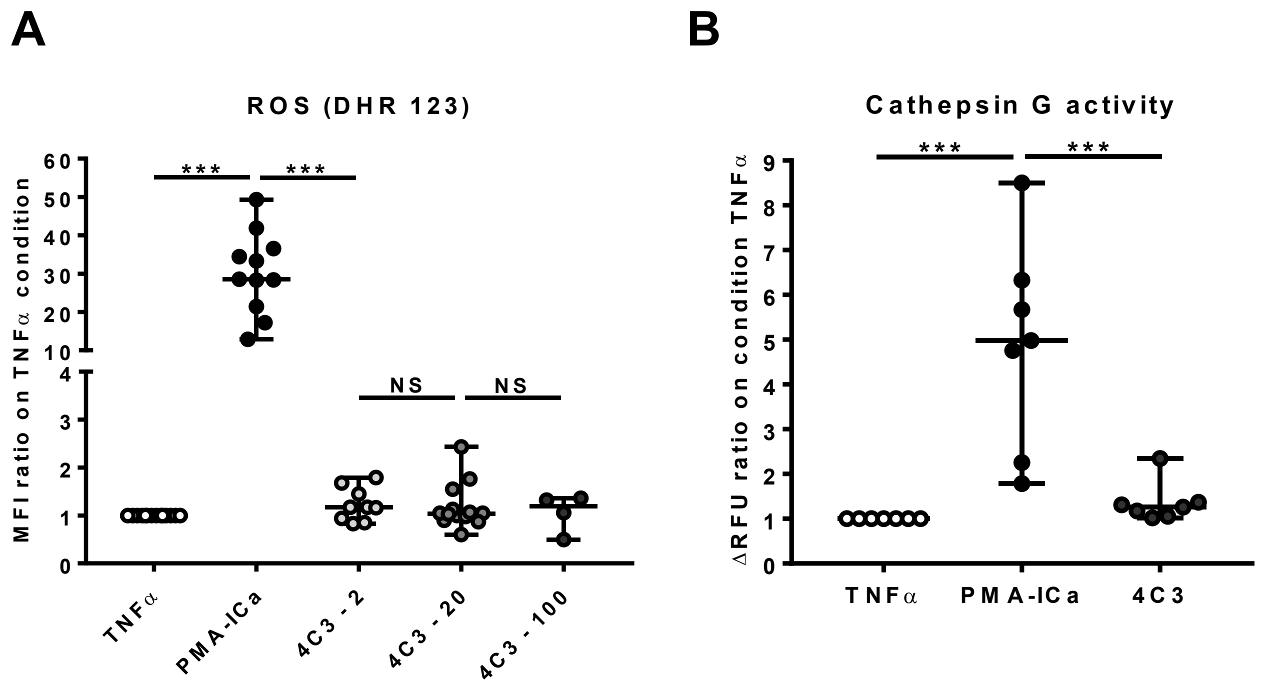

Supplementary figure 4: 4C3 does not induce production of ROS and is not able to increase cathepsin G activity in neutrophils. Purified neutrophils from 11 independent healthy donors were primed with TNFα (2 ng / ml) for 15 minutes at 37°C (white circles) before incubation for 45 minutes with PMA-ICa (black circles) or 4C3 (grey circles). **(A) 4C3 does not induce production of ROS whatever the concentration used.** Different concentrations (2, 20 and 100 µg / ml) of 4C3 were used and ROS production was assessed by measuring the fluorescence (MFI) of DHR 123 using flow cytometry. Results are expressed in MFI ratio compared to normalized TNFα condition. n=11 for all conditions except for 100 µg / ml with n=4. NS: Non-significant ; ****$p<0.0005$*. **(B) 4C3 does not increase cathepsin G activity of neutrophils.** Cathepsin G activity was assessed after adding the substrate to the supernatants of neutrophils from 7 independent healthy donors and reading the fluorescence by spectrofluorimetry. Results are expressed in ΔRFU ratio compared to normalized TNFα condition. n=7. ****$p<0.0005$*.

Supplementary Figure 5

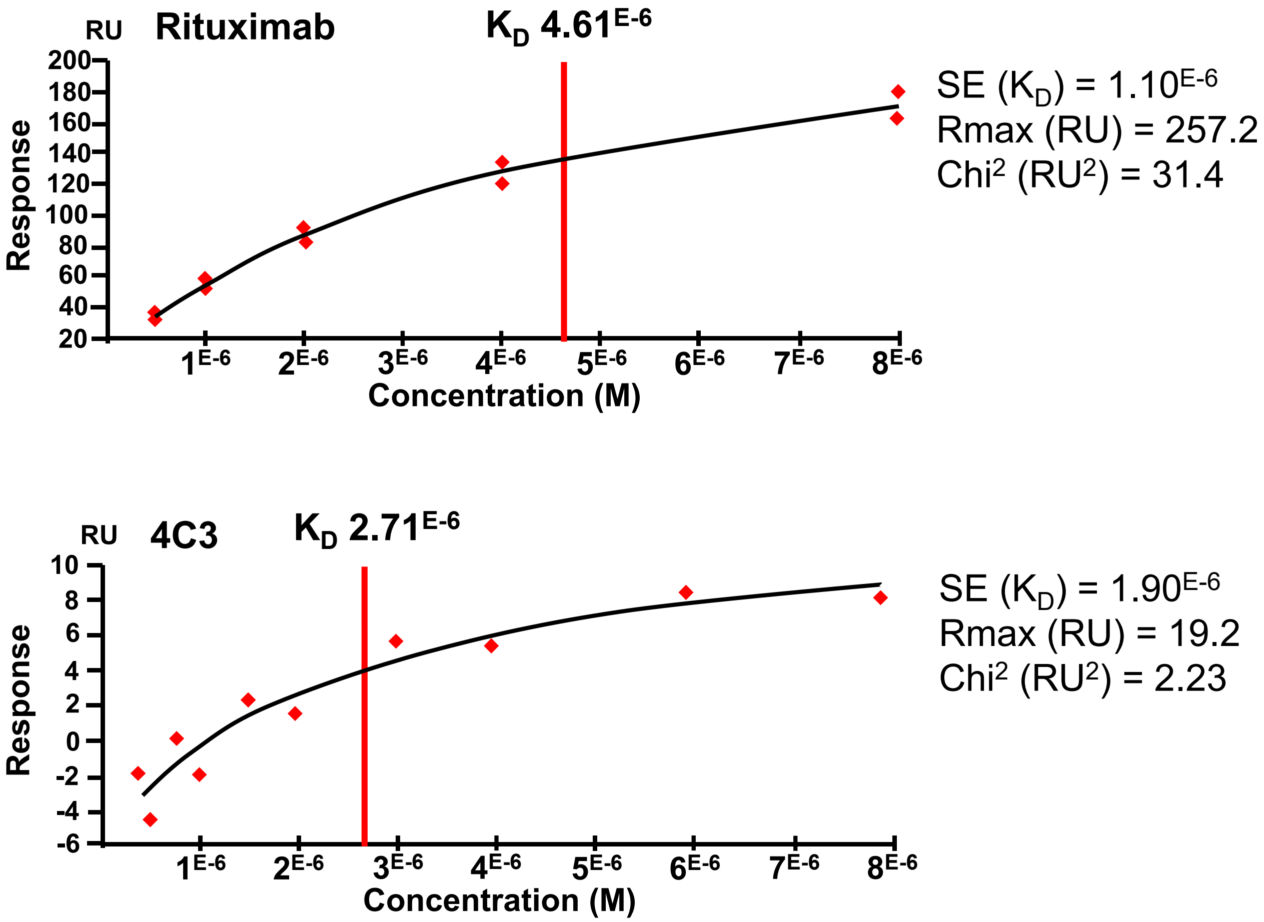

Supplementary Figure 5 : 4C3 is able to bind FcγRIIIB. Affinity measurement of 4C3 for FcγRIIIB (CD16b) was assessed by SPR using poly-histidine FcγRIIIB captured on an immobilised anti-poly-histidine mAb. Binding was monitored as an increase in SPR signal expressed in Resonance Units (RU). Affinity was evaluated as a steady-state equilibrium fitting model. Rituximab (upper panel) was used as a control for FcγRIIIB binding. The K_D value obtained is indicated on the graph with the red line. SE: Standard Error. Rmax is the saturation signal.
