# Supplementary material for: 4C3 Human Monoclonal Antibody: A Proof of Concept for Non-pathogenic Proteinase 3 Anti-neutrophil Cytoplasmic Antibodies in Granulomatosis With Polyangiitis
Source: Front Immunol. 2020 Sep 25;11:573040. doi: 10.3389/fimmu.2020.573040 (PMC7546423; doi:10.3389/fimmu.2020.573040)
Supplement: Supplementary file 2 [file Data_Sheet_2.PDF]

Supplementary table 1. Affinity of 4C3 and r4C3 for PR3 measured by Surface Plasmon Resonance.

	k_a (1 / Ms)	k_d (1 / s)	K_D (M)	Rmax (RU)	Concentration (M)	Chi^2 (RU^2)	U-value
**4C3**	1.26E+07	0.00936	**7.41E-10**			0.723	3
Cycle 5				78.35	1.25E-09		
Cycle 6				76.5	2.50E-09		
Cycle 7				80.18	5.00E-09		
Cycle 8				71.98	1.00E-08		
Cycle 9				75.01	2.00E-08		
**r4C3**	1.37E+07	0.009358	**6.84E-10**			1.06	4
Cycle 14				161.8	1.25E-09		
Cycle 15				128.1	2.50E-09		
Cycle 16				110.9	5.00E-09		
Cycle 17				98.36	1.00E-08		
Cycle 18				100.5	2.00E-08		
